# The Involvement of Caregivers in the End-of-life Care of an Older Adult Living in a Long-term Care Home: A Qualitative Case Study with Nurses and Relatives

**DOI:** 10.1177/08445621241247862

**Published:** 2024-04-15

**Authors:** Isabelle Auclair, Anne Bourbonnais

**Affiliations:** 1 PhD candidate, Faculty of Nursing, Université de Montréal, Montreal, Canada; 2 Research assistant, Research Centre of the Institut universitaire de gériatrie de Montréal, Montreal, Canda; 3 Full professor, Faculty of Nursing, Université de Montréal, Montreal, Canada; 4 Researcher, Research Centre of the Institut universitaire de gériatrie de Montréal, Montreal, Canada; 5 Chairholder of the Canada Research Chair in Care for Older People Chairholder of the Research Chair in Nursing Care for Older People and their Families, Montreal, Canada

**Keywords:** Terminal care, informal caregivers, family, aged, nursing

## Abstract

**Background:**

A key role of nurses working in long-term care homes (LTCHs) is to promote the involvement of care partners in end-of-life (EOL) care. However, studies on the involvement of care partners in EOL care in LTCHs have focused on care planning and decision-making. While care partners can participate in other ways, it's unclear how they are currently involved in EOL care by staff.

**Purpose:**

We aimed to explore the involvement of care partners in the EOL care of an older adult living in a LTCH.

**Methods:**

A qualitative case study was conducted. Data was collected from a sample of four nurses and three care partners, using sociodemographic questionnaires, individual semi-structured interviews, documents pertaining to the LTCH's philosophy for EOL care, and a field diary.

**Results:**

The results of a thematic analysis showed the broad scope of care partners’ possible involvement, including contributing to care, obtaining information, and being present. As there was some variation in care partners’ desire to be involved, nurses seemed to rely on them to convey their wishes. To promote this involvement, some strategies aimed at health professionals and managers were suggested.

**Conclusions:**

These results can guide improvement in clinical practices and raise awareness on the EOL care experiences of care partners.

## Background & purpose

Deaths are increasing worldwide due to population ageing (Cheng et al., 2020), indicating the importance of ensuring quality palliative and end-of-life (EOL) care for older adults in healthcare settings. Palliative care has been recently redefined by experts as holistic care intended to relieve the multifaceted suffering (physical, social, spiritual, emotional) of people living with severe illnesses, including those with a high risk of mortality (Radbruch et al., 2020). While EOL care has the same aim, it is often specific to care provided when death is imminent (Froggatt & Payne, 2006; Galantin et al., 2019).

EOL care is especially a priority in long-term care homes (LTCHs), where it is estimated that about one in three older adults die each year (Vossius et al., 2018). In LTCHs, EOL is associated with suffering in both the older adults and their care partners. We define a care partner as a person who has an emotional bond with the older adult, which can be a family member or a friend. They all can face psychological difficulties, such as not being prepared for the oncoming death (Cagle et al., 2017). EOL can also be characterized by pain, dyspnea, or other uncomfortable symptoms for the older adult (De Roo et al., 2015; Pivodic et al., 2018). Involving care partners is a way for health professionals to lessen the suffering experienced by the older adults and their care partners during EOL and optimize care in LTCHs.

Older adults can benefit from having their care partners involved in EOL care, especially when they are unable to clearly communicate their preferences and needs (Galantin et al., 2019). In this situation, care partners can advocate for older adults to ensure quality of care or respect of their wishes (Bollig et al., 2016; Lopez et al., 2013; Mulqueen & Coffey, 2017). Moreover, care partners in LTCHs can also benefit from their involvement in EOL care. Being involved, by receiving information (e.g., on EOL symptoms), helps some care partners have a sense of control in a situation that can be stressful (Church et al., 2016). In addition, explaining the disease trajectory to care partners can contribute to their preparedness for death (McCleary et al., 2018). Some care partners also experience positive psychological effects from being involved, such as feeling valued and having a higher self-esteem (Ducharme, 2012; Lopez et al., 2013).

However, there are also difficulties associated with care partners’ involvement in the EOL care of older adults in LTCHs (Bollig et al., 2016; Church et al., 2016; Goddard et al., 2013; Romøren et al., 2016). Professionals can feel frustrated and find communication with care partners stressful when the latter change care decisions or require hospitalization when death becomes imminent (Goddard et al., 2013). In addition, care partners designated as proxies in EOL care decisions can experience this responsibility as a burden (Bollig et al., 2016; Church et al., 2016).

While the literature on the involvement of care partners in the EOL care of older adults in LTCHs points out both benefits and difficulties, providing support to care partners and addressing their needs is a fundamental component of health professionals’ practices (Radbruch et al., 2020). Therefore, the involvement of care partners should be an integral part of nurses’ practice in LTCHs.

To define this central concept of involvement of care partners in EOL care, we used Andershed and Ternestedt (2001)'s theory which describes three possible types of involvement: 1) information received or gathered (to know); 2) presence and interest in the dying person (to be); 3) participation in tasks or care (to do). Information is described as key in empowering informal care partners to know how they want to be involved in care and act on these wishes.

Based on a literature review (*n* = 24) supported by Andershed and Ternestedt (2001)'s theory, studies offer only a superficial glance at the involvement of care partners in the EOL care of older adults in LTCHs (Auclair & Bourbonnais, 2020). This involvement is almost exclusively described in terms of care planning and decision-making (Ampe et al., 2016; Biola et al., 2010; Bollig et al., 2015; Dreyer et al., 2009; Gjerberg et al., 2011, 2015; Kirsebom et al., 2017; Masukwedza et al., 2019; Percival & Johnson, 2013; Romøren et al., 2016; Sarabia-Cobo et al., 2016; Thoresen & Lillemoen, 2016; van Soest-Poortvliet et al., 2014). Only one descriptive correlational study explored other ways care partners are involved in care (e.g., monitoring, personal hygiene, meals). However, it focused on the last month of life (Williams et al., 2012) and not on the last days or hours. As such, there is a clear gap in knowledge about the last days and hours in LTCHs, when daily functioning is interrupted by a significant decline in energy, leaving the older adult unable to stay awake, communicate, or even eat (Centre intégré universitaire de santé et de services sociaux de la Capitale-Nationale, 2015). A recent literature review on family involvement in residential long-term care, including LTCHs, further confirms the need for more descriptive research on family involvement when transitioning to EOL care to inform the development of future interventions (Gaugler & Mitchell, 2022). This need for knowledge should extend to the involvement of care partners when death becomes imminent.

Furthermore, care partners’ desire for involvement is addressed only cursorily in two studies, with brief comments in the results section (Gjerberg et al., 2015; Lopez et al., 2013). Finally, no study was found on strategies promoting the involvement of care partners during the last days and hours of older adults living in LTCHs.

Our aim was to explore the perspectives of care partners and nurses on the involvement of care partners in the EOL care (last days and hours of life) of an older adult living in a LTCH. Our research questions were as follows:

1. How are care partners involved in the EOL care of an older adult in a LTCH?
2. How do care partners wish to be involved in the EOL care of an older adult in a LTCH?
3. What strategies can promote the involvement of care partners in the EOL care of an older adult in a LTCH?

## Methods and procedures

### Design

We conducted a qualitative case study of the collective instrumental type (Stake, 1995). We defined a case as a care partner or a nurse who is or was involved in the EOL care of an older adult living in a LTCH. This design was considered particularly relevant as it considers the underlying context of the cases studied, here the context of the LTCH (Stake, 1995). Andershed and Ternestedt (2001)'s theory was used as a framework to collect and analyse data.

### Sample

The selection of cases is an important part of the methodological process in case studies, as it must contribute to furthering understanding of the phenomenon (Stake, 1995). We recruited the participants from a public LTCH of approximately 130 beds in Montreal (Quebec, Canada). A director of the integrated health care institution regrouping 17 LTCHs helped us identify a typical LTCH, with no distinguishing expertise in EOL care. We intended for our results to be more transferable to other LTCHs. We selected participants with different sociodemographic characteristics (e.g., gender), through a purposive sampling strategy, to obtain different perspectives on the studied phenomenon. The LTCH managers helped with recruitment by approving the display of posters detailing the study and by referring the first author to potential participants and informants who could help identify care partners meeting the selection criteria for the study. Furthermore, the first author directly recruited nurses by visiting the LTCH and enquiring about their interest in the project.

The inclusion criterion for care partners was that they should view themselves as the primary caregiver of an older adult currently experiencing their last days of life or who had passed away in the last month before recruitment (to minimize memory bias). As for the nurses, they needed to have provided EOL care to an older adult and to have at least six months of experience in the LTCH. All participants had to be fluent in French or English and be aged 18 or older.

In a collective case study, the sample size is typically small. The recommendation is to recruit around three to five cases to focus on in-depth data collection (Schoch, 2020; Stake, 2006). Based on this criterion, three care partners and four nurses (*n* = 7) were recruited from one LTCH in Montréal (Canada).

### Data collection

The first author collected data in 2020 from February and March through individual semi-structured interviews, sociodemographic questionnaires, documents pertaining to the LTCH's philosophy for EOL care, and a field diary. She conducted most interviews in the LTCH, but two participants preferred other settings (i.e., home, library). Interviews were recorded and lasted on average 51 min (34 to 78 min). She had some experience with conducting qualitative interviews and was coached by AB beforehand. She also used an interview guide (one version for nurses and one for care partners) covering each research question and based on the three categories of involvement described by Andershed and Ternestedt (2001). The interview guide included a preliminary statement to explain the interview process and to contextualize EOL care in the last days and hours. The first question was general to put participants at ease, but most questions followed up based on what the nurse or care partner had said. An example of a question related to the first research question (actual involvement of care partners) is: “Could you tell me about a situation in which you cared for an older adult in the last days of their life?”, “In what contexts did you interact with care partners?”. For the second research question (wishes for the involvement of care partners), a question was: “Do you wish you had been involved in care differently, either less or more?”. And for the third research question (strategies to promote the involvement of care partners), examples of questions are: “How can professionals better involve care partners in EOL care? If necessary, explore *to be* (e.g., encourage visits), *to know* (e.g., increase knowledge), *to do* (e.g., encourage participation in tasks/care)”, “What would facilitate your involvement?”, “What barriers hindered the involvement of care partners?”

As for documents, IA considered them relevant when they had contextual data on the LTCH's philosophy for EOL care. In total, eight documents were included (e.g., booklet on EOL care for older adults and their care partners, EOL procedures for health care professionals, facility's policies and code of ethics at the EOL). Finally, in her field diary, she documented her experience (informal conversations, observations of the environment and behaviors, and feelings) and thoughts on possible personal bias when visiting the LTCH and interviewing participants.

### Data analysis

Data was analyzed by IA in the Excel software using an interpretative thematic analysis (Braun & Clarke, 2006) and then was reviewed by AB. This method included six steps. The first step involved becoming familiar with the data by transcribing and reading the verbatim interviews, the entries in the field diary, and the relevant texts in the documents. During this step, initial ideas were written down. The second step consisted in generating initial codes. To organize the codes and preserve the context from which they were generated, we added prefixes and suffixes about the data type (interviews, documents, or field diary), participant type (nurse or care partner), and research question the code was related to. Table 1 gives an example of the coding process, with transcription and codes translated from French to English. The third step involved a search for themes based on the codes that were organized into a tree structure. Preliminary themes emerged from this organization of codes. The fourth step was reviewing those preliminary themes. To do so, we ensured that the grouping of codes was consistent within and between themes and that their organization was in line with the original context from which the codes were generated. In the fifth step, themes were defined and named, their scope established, and subthemes added. Finally, the last step involved producing the report and including verbatim transcripts to support the results.

**Table 1.** Step 2 of data analysis: generating initial codes.

Verbatim	Codes
Interviewer: Are there other difficulties with the family that sometimes hinder involvement?	
Nurse: Yes. There will be families present, but also too present. “Don't do it, don't do it like that. No why did you inject him? Why are you giving it to him? But I'm not sure you should give it to him. I'll call my sister-in-law who's a nurse to find out…My cousin, my niece, is a nurse. I want to know what she thinks, she's just out of school.”	S-Influencing Factors (Disadvantages)- Care Partners are seen as too present when they question the care provided by nurses-N A-Care (Care Partners are involved)- Care Partners criticize the care provided (nature, execution)-N A-Knowledge (Care Partners are involved)-Care Partners question reasons for medication administration-N

Prefix

S: Strategies to promote the involvement of care partners

A: Actual involvement of care partners

Suffix

N: Nurses

### Ethical considerations

Our project was approved by the institutional review board of the LTCH (CER VN 19-20-45). Informed consent was obtained from participants. Considering the topic's sensitivity, a list of psychological resources was given to care partners and a follow-up call was carried out 48 h after the interviews to inquire about their well-being. Care partners conveyed no distress during this follow-up. If the nurses had shown distress, they would have been referred to their Employee Assistance Program. This was not necessary as nurses were comfortable discussing their experience of providing EOL care.

## Results

### EOL care documents

The LTCH's EOL philosophy underlying documents collected is based on a caring approach and a partnership with the older adult and, in some cases, their family.

The documents for health professionals address screening for EOL signs and symptoms, as well as the physical care and psychological care that should be provided when the older adult is considered at the EOL. Rounds are to be conducted every 30 min, whether by nursing assistants or nurses, to monitor the presence of suffering. In addition, the LTCH offers weekly medical rounds where the physician meets with older adults and their care partners, regardless of EOL status. A nurse who is an expert in EOL care and who works in several LTCHs to ensure that this care runs smoothly is also available.

Upon admission to the LTCH, an information packet is to be given to older adults and their care partners. This information packet explains EOL terminology, how to recognize and relieve EOL signs and symptoms, what interventions to avoid, and possible EOL trajectories. An interprofessional meeting is scheduled each year, in addition to when health deteriorates, to review the older adult's level of care. Once the older adult is recognized as being at the EOL, a cart of comfort items (e.g., moisturizer, essential oils, ambient music) is made available to care partners, if desired.

To optimize services, an EOL care committee with various staff members (e.g., managers, housekeeping staff, spiritual counselor, nurses) has been established. This committee reviews the services provided through a form completed by care partners following the death of an older adult in the LTCH.

### Participant characteristics

Despite nursing generally having a higher proportion of women, there are both men and women represented in our study. Nurses all worked full-time on the day shift, but two also worked on the evening and night shifts, offering insight on the involvement of care partners in different working conditions. Although most nurses had few years of experience working in LTCHs, they stated having extensive experience with EOL. Only one nurse did not pursue a bachelor's degree following a technical diploma in nursing.

As for the care partners, two were women and one a man, and they were either a friend or a child of an older adult deceased in the LTCH. Care partners had at least a college degree, but their marital and employment status differed. They visited an older adult many times per week, with visit lengths varying from 30 min to half a day. Care partners were involved in the EOL care of very old adults, living with or without a major neurocognitive disorder, recently admitted to the LTCH. Sociodemographic data on the participants are detailed in Table 2.

**Table 2.** Sociodemographic characteristics (*n* = 7)

Sociodemographic Characteristic	Nurses (*n* = 4)	Care Partners (*n* = 3)	Older Adults (*n* = 3), as Reported by Care Partners
Age in years (M ± SD) [range]	33.5 ± 6.2 [27–41]	62.7 ± 10.8 [55–75]	81.7 ± 4.6 [79–87]
**Gender**			
Women	2	2	1
Men	2	1	2
**Ethnicity**			
Caucasian	-	3	-
**Marital status**			
Married / common-law partner	-	1	-
Divorced	-	1	-
Single		1	-
**Employment status**			
Full-time	4	1	-
Part-time	-	1	-
Retired	-	1	-
**Education**			
Technical diploma	1	2	-
Degree in progress	2	0	-
Degree	1	0	-
Master's	0	1	-
**Working shifts**			
Day	2	-	-
Evening	0	-	-
Night	0	-	-
Rotation	2	-	-
**Years working as a nurse** (M ± SD) [range]	6.2 ± 4.2 [2–12]	-	-
**Years working as a nurse in long-term care homes** (M ± SD) [range]	4.5 ± 1.7 [2–6]	-	-
**Experience with end-of-life care**			
Very little	0	-	-
Little	0	-	-
Moderate	1	-	-
Extensive	3	-	-
Very extensive	0	-	-
**End-of-life continuing educatio**n			
Yes	4	-	-
No	0	-	-
**End-of-life continuing education addressing the involvement of care partner**s			
Yes	3	-	-
No	1	-	-
**Nature of relationship with older adult**			
Spouse	-	0	-
Son/Daughter	-	1	-
Sibling	-	0	-
Nephew/Niece	-	0	-
Friend	-	2	-
**Quality of relation with the older adult**			
Poor	-	0	-
Fine	-	1	-
Good	-	0	-
Very good	-	1	-
Excellent	-	1	-
**Frequency of visits**			
Every day	-	0	-
Several times a week	-	3	-
At least once a week	-	0	-
Several times a month	-	0	-
**Duration of visits**			
30 min	-	1	-
1–2 h	-	1	-
More than 2 h	-	0	-
Half-day	-	1	-
All day (not including night)	-	0	-
**Time spent living in long-term care home**			
Less than a month	-	-	1
1 to 6 months	-	-	1
1 year	-	-	1
2 to 5 years	-	-	0
**Dementia**			
Yes	-	-	2
No	-	-	1

### Description of involvement and its influencing factors

We identified two themes describing the nature of the possible involvement of care partners in EOL care in LTCHs and the influencing factors that can act as facilitators or barriers. Quotes have been translated from the original French.

### The involvement of care partners in EOL care: Variable and vast

This theme addresses the scope of care partners’ involvement in the EOL care of an older adult living in a LTCH, including how care partners can involve themselves and be involved by staff. The nature of this involvement has been organized according to the three categories of Andershed and Ternestedt (2001), namely, to know, to be, and to do, and is summarized in Table 3 following a description of the subthemes.

**Table 3.** Scope of care partners’ involvement in the EOL care of an older adult living in a LTCH

	**Care Partners Involved Themselves**	**Health Professionals Involved Care Partners**
**To know**	Asked questions about health status interventions provided or not end-of-life process Asked other people for their opinions on care provided	Offered follow-ups on health status Offered follow-ups on interventions provided or not to the older adult: activities of daily living medication psychological support interventions to avoid reasons for carrying out interventions, or not effects of interventions Gave education about end-of-life: signs and symptoms myths related to opiates Communicated information from other professionals Answered questions
**To be**	Visited the older adult in the long-term care home Communicated with the older adult: Verbally o through physical contact	Promoted care partners’ presence at the bedside explicitly (e.g., when they informed care partners of a health decline) o implicitly (e.g., when they shared with care partners the benefits of their presence)
**To do**	Participated in care planning and decision-making to: change the level of care accept or refuse specific interventions Helped with activities of daily living Asked health professionals to perform activities of daily living Monitored: declines in health status end-of-life signs and symptoms execution of care efficacity of medication Asked for medication to be administered Brought objects to the long-term care home	Encouraged participation in: care planning and decision-making direct care Supported participation in activities of daily living explained how care should be provided

#### Informed by the Care Team, but Not Always Adequately

Participants said that the interprofessional team could involve care partners by informing them about various aspects of EOL. Both the care partners and nurses emphasized how important it was for care partners to be briefed when a deterioration occurred in the older adult's health. Acknowledging declines in health was described as a way to anticipate oncoming death and, thus, was considered essential information for nurses to share with care partners so they could be involved as they wanted in the last moments of the older adult's life. Some nurses shared that they systematically gave updates about evolutions in health decline, at times because care partners initiated the conversation.

Nurses also involved care partners by explaining what interventions were or were not carried out in the older adult's final days, along with describing the reasons and the effects. These interventions included helping in activities of daily living (ADL), giving medication, monitoring EOL signs and symptoms, and offering psychological support to the older adult when the care partners were absent. Moreover, nurses discussed interventions to avoid during EOL (e.g., hospital transfers, administration of oxygen, artificial hydration and feeding) with care partners. However, according to participants, some care partners did not feel the need to be informed about interventions, as they considered it to fall under the expertise of health professionals. In contrast, the experience of one care partner showed how information can be too superficial compared to what was hoped for:

*I said, “He didn’t eat or drink anything, are you going to feed him artificially?” They said, “No, you realize that when you are in end-of-life care, we don't do anything, we let him die.’’ I said, “I didn't know end-of-life care is letting people die. I still thought you could give him some water to quench his thirst.” No nothing. They said that and they left.” (Care partner 3, lines 34–35)*

One nurse mentioned how some care partners educated themselves through means other than asking staff questions. For example, they might have searched the Internet or questioned acquaintances who have health care expertise.

Yet, all participating nurses stated that one of their key roles regarding the involvement of care partners during EOL was education about the EOL process, including the signs and symptoms associated with EOL. For example, when care partners refused opiate injections out of fear of accelerating death, they would explain how this is a myth. Nonetheless, our results show that health professionals could also give inaccurate information when they lacked knowledge, such as communicating as a fact this myth about opiates accelerating death.

Furthermore, collaboration between staff members emerged as an important factor in informing care partners, as it helped answer their questions about care and the health status of the older adult. Nurses could also inform or remind care partners that other important members of the interprofessional team were available, such as the social worker and the psychologist. They also referred care partners to a spiritual counselor to help them cope with the onset of death and to meet their spiritual needs. One care partner recalled meeting the spiritual counselor, who gave her his contact information.

#### Ensuring a Presence for the Older Adult

Care partners were present in many ways for the older adult receiving EOL care. They often visited more frequently than before the EOL. Even care partners who had rarely been to the LTCH were often present during the final moments. The duration of visits varied: some care partners wanted to be with the older adult as much as possible, while others found it emotionally difficult. Care partners could also be present by giving special attention to the older adult to increase their comfort, for instance by bringing objects to personalize the room. Some were present through verbal and nonverbal communication. Nurses mentioned playing a role in implicitly encouraging the presence of care partners by stating the benefits for the older adult. Nurses also reported that they provided an “EOL cart” designed for the comfort of older adults that could be used by care partners. The EOL cart contained items like moisturizing cream for massages, essential oils, and a player for ambient music.

In situations where older adults had no family ties or where their care partners’ lacked availability, nurses sometimes organized for other people, such as volunteers, to be present. Moreover, the LTCH's staff and other residents were sometimes considered as the older adult's family and ensured a presence.

#### A Lot More Than Care Planning and Decision-making

Participants shared how care partners got involved in care, including but not limited to, planning and decision-making. Regarding the latter, the care partners of older adults considered unable to give consent were involved as surrogates in changing the level of care as well as in accepting or refusing interventions provided by health professionals during the EOL. When the older adult could participate in care planning and decision-making, participants described that some care partners were not involved because they believed it was up to the older adult to choose or because the latter did not want them involved.

As for other types of care provided during the last days and hours, care partners could be involved in ADL, such as mobilization, personal hygiene, and mouth care. Nurses shared how they could support the involvement of care partners by explaining how to perform the care appropriately. However, according to participants, many care partners did not want to participate in this type of care, especially in personal hygiene care. The care partners who participated in ADL before the EOL were usually the ones who continued doing so during EOL. Professionals could be apprehensive about asking care partners to be involved in this type of care. One nurse explained that it required tact but could promote the involvement of care partners:

*They [nursing assistants] should ask the family to help them but, at the same time, we say to ourselves, “If I tell the family to help me, how will they perceive it? […] Because [care partners could think] ‘you want us to do your job.’” But apart from this explanation, it takes, let's say, pedagogy to tell the person, “I'm not asking you because I want you to do my job, but if you feel the need to participate in care, it will also be helpful.” It's how you describe the offer that matters. […] The family will have the right arguments to say yes or no […]. They will even give the limits of their participation, but if we don't ask them, what is not said is not established. (Nurse 3, lines 169–171)*

Care partners could also be involved indirectly, by asking staff to provide care and monitoring its quality. Some contributed to pain or discomfort management by monitoring the EOL signs and symptoms experienced by the older adult. This kind of involvement could be initiated by care partners or nurses. Although care partners could request medication when they thought the older adult had discomfort, our results showed that its administration was viewed as a health professional's role, as it requires medical knowledge.

### The influence of personal and organizational conditions in the involvement of care partners

This theme presents conditions influencing the involvement of care partners in EOL care, from their wishes to their individual, relational, and organizational characteristics. These results are summarized in Table 4.

**Table 4.** Factors and supporting strategies influencing the involvement of care partners in the EOL care of an older adult living in a LTCH

**Factors Influencing the Involvement of Care Partners**	**Strategies Supporting the Involvement of Care Partners with Respect to Their Wishes**
**Individual** Health professionals *(**facilitators**)* Perception of being caring by involving care partners Openness to the involvement of care partners Knowledge on end-of-life care Health professionals *(**barriers**)* Lack of follow-up between professionals Perception of breaching a resident's right to confidentiality Discomfort with end-of life care Care partners (**facilitators**) Culture valuing being present when a relative is ill Explicitly manifesting their interest in being involved in care Care partners (**barriers**) Perception that being involved in care is bothersome to health professionals Emotional and physical difficulties Personal obligations	**Carried Out by Professionals** Normalized the involvement of care partners in its various forms Avoided judgements on the involvement of care partners Explicitly offered the possibility of participating in end-of-life care Adopted a caring approach with care partners and older adults: positive attitude empathy personalization of care interest toward the end-of-life experience **Carried Out by Managers** Offered regular continuing education on end-of-life care Encouraged professionals to support each other in developing their knowledge about end-of-life care
**Relational** Expression of wish from older adults that their care partners be involved in care (***facilitator***) Lack of a trusting relationship between care partners and professionals (***barrier***)	
**Organizational** Heavy workload of professionals (***barrier***)	

#### Personalizing Involvement According to Care Partners’ Wishes

Analysis of the data from both types of participants shows that the wishes of care partners regarding their involvement were unique. They varied from care partners not wanting to be involved at all to wishing to be involved as much as possible. Care partners occasionally expressed their wishes for involvement, allowing nurses to personalize EOL care accordingly. When involvement preferences were not stated explicitly by care partners, nurses tried to deduce them by paying attention to their behaviors. They associated behaviors such as asking questions or staying in the room when care was being provided as a desire to be involved, while the opposite behaviors were viewed as not wishing to be involved. Although some nurses relied on inferences, the following account highlights how they should be active in promoting the involvement of care partners:

*Most of the time, we observe that it's the family that involves itself, that initiates. […] “I want to provide the care, I want to do this, I want to do that” [care partners say]. It would be good if it was us [nurses] who suggest it to them, because sometimes with this ordeal, it's more difficult. […] Probably that they [care partners] would have wanted to get involved, but the fact that they are a little upset, a little shaken [prevents them from doing so]. (Nurse 2, line 46)*

Nurses described situations when they prompted the involvement of care partners by asking if they wanted to participate in EOL care, in an effort to offer them an opportunity to express their involvement wishes. They did not ask care partners about their desire to take part in specific care or their need for precise information.

#### Being Involved: Many Barriers and Facilitators but Very Few Supporting Strategies

Many factors influenced the involvement of care partners in EOL care. The individual characteristics of health professionals and care partners could either hinder or promote involvement. Nurses who were open toward the involvement of care partners and perceived it as a form of caring could facilitate such involvement. This openness can be translated into behaviors that help care partners feel comfortable participating in care, such as nurses normalizing or avoiding any judgments about how care partners choose to get involved in EOL care. By contrast, involvement could be hindered by nurses who were uncomfortable discussing EOL with care partners or who had limited knowledge about EOL care or about deontological rules regarding sharing information. These barriers can be decreased by managers ensuring continuous education on EOL care or fostering expertise sharing between colleagues. Moreover, inadequate follow-up between health professionals about the care of the older adult (e.g., during shift changes, miscommunication between colleagues in different roles) was also a barrier to care partners’ involvement. They sometimes had difficulty answering care partners’ questions or providing updates on the resident's health and care.

In terms of the individual characteristics of care partners, cultural background may play a role in promoting involvement. For example, some cultures value being present when a care partner is ill:

There is a lady who died, her daughter in the end she wanted her son to come see his grandmother […]. In our African values, kinship is extremely important. These are times when it is extremely important to have the family around if possible. (Nurse 3, line 55)

Nevertheless, our results mainly indicate characteristics in care partners that can hinder their involvement. Some care partners thought asking questions or inquiring about participating in care would be bothersome to professionals and thus restricted their own involvement. In other cases, care partners had limited knowledge about their potential scope for involvement. Care partners could also lack the physical or psychological capacity to be involved as they wished. For example, they might be unable to help in ADL due to emotional difficulties or physical strength limits. Furthermore, the presence of care partners with the older adult can depend on their ability to take time off from work.

Relational aspects also impacted the involvement of care partners. When a trust in health professionals was lacking, some care partners increased their involvement to ensure the quality of EOL care and adopted a more confrontational attitude toward staff to advocate for the well-being of the older adult. Results show how a trusting relationship could develop when nurses and other health professionals providing EOL care used a caring approach with care partners as well as with the older adults, by conveying empathy, having a positive attitude, personalizing care, and being interested in the EOL experience. Finally, care partners identified the staff's heavy workload and lack of time as an organizational barrier to their involvement.

## Discussion

This study explored the perceptions of care partners and nurses on the involvement of care partners during the EOL (last days and hours) of an older adult living in a LTCH, including how they were involved, how they wished to be involved, and the strategies used to promote involvement. Regarding care partners’ ways of being involved, the results show they can be involved through information obtained on the health status, on the nature and effects of interventions, and on the EOL process and care. Sharing such information is important as it helps care partners understand the reasons underlying the care provided and better anticipate the coming death. The concrete ways identified for increasing care partners’ knowledge during the last days and hours add to what is documented in the literature, which has focused on care planning and decision-making (Dreyer et al., 2009; Gjerberg et al., 2011; Kirsebom et al., 2017; Percival & Johnson, 2013; Sarabia-Cobo et al., 2016). Thus, results can guide health professionals in educating care partners during EOL. In fact, one of nurses’ key roles in EOL care, identified in a qualitative metasynthesis across health settings, is supporting care partners by educating them on the health situation and personalizing this information according to their understanding (Sekse et al., 2018). Our results further support the importance of nurses playing an informative role, specifically during EOL in LTCHs, where they work closely with care partners, and doing so appropriately without giving inaccurate information.

Our findings also highlight that care partners usually become more involved, in terms of visits, when faced with the oncoming death of the older adult. Similarly, a longitudinal descriptive study over 90 days noted an increase in care partners’ visits of residents with major neurocognitive disorders in a specialized LTCH, with about 1 in 3 (31%, *n* = 16) daily visits in the last month (day 30 to day 8 before death), as compared to 3 in 4 care partners (75%, *n* = 41) visiting daily during the last week of life (Koppitz et al., 2015). However, this study does not provide results on the reasons why some care partners visit more often than others. Our study describes factors that can help understand what influences this type of involvement by care partners, such as their psychological well-being and their personal obligations outside the LTCH. Moreover, our results show that nurses can ensure that someone is present at EOL for an older adult who receives no visits from care partners. A specificity of care in LTCHs is how residents develop strong relationships over time with staff as well as with other residents and their family members (Bourbonnais et al., 2021a; Canham et al., 2017). Therefore, nurses can consider these relationships when planning for the EOL care of residents with no care partners outside the LTCH.

While our study shows some aspects of how care partners can be present and contribute to the psychosocial care of the older adult through massages and a relaxing ambiance, it shows a limited understanding of their contribution in spiritual care during the last days and hours. This could be explained by the fact that care partners did not identify with any religion or spirituality and did not request that for the LTCH to provide specific rites or rituals. We did not ask about the religion and/or spirituality of care partners in our sociodemographic questionnaire, but this should be considered in further studies addressing their involvement in EOL care. However, spiritual care in the EOL is more comprehensive than just faith and religion, and can include meaning, acceptance, hope, or reconciliation (García-Navarro et al., 2022; Koper et al., 2019). Nurses can be important actors in promoting the involvement of care partners in these aspects, but there seems to be a narrower view of their role as participants only referred to the spiritual counselor.

The literature on the involvement of care partners in EOL care appears mainly limited to advance directives and advance care planning, and is not specific to the last days and hours (Ampe et al., 2016; Biola et al., 2010; Bollig et al., 2015; Dreyer et al., 2009; Gjerberg et al., 2015; Kirsebom et al., 2017; Masukwedza et al., 2019; Percival & Johnson, 2013; Romøren et al., 2016; Thoresen & Lillemoen, 2016; van Soest-Poortvliet et al., 2014). Regarding the last month of life, Williams et al. (2012) mention that care partners are involved in ADLs, such as hygiene. Our results expand on the description of the involvement of care partners in ADLs by noting that in the last days and hours of life, they sometimes perform mouth care and mobilize the older adult. This care is described as generally undertaken by care partners who remain involved in ADLs throughout the older adult's stay in a LTCH. Care partners can also participate in discomfort management. In LTCHs, there appears to be only limited possibility of involvement in care that is not part of the usual nursing assistant role, such as the administration of medication. Empirical evidence suggests that care partners are involved in this type of care in the context of home care because of the absence of professionals at the bedside during EOL and that this responsibility generates mixed feelings (Wilson et al., 2018). Some care partners are afraid of under- or overmedicating and may feel powerless in managing EOL symptoms or, quite the opposite, feel empowered by it (Wilson et al., 2018). Further studies on the openness and the ethical implications for care partners and health professionals of the former's involvement in care is relevant to better understand the potential challenges to that involvement in LTCHs or other care facilities.

In summary of the ways care partners are involved in the EOL care of older adults in LTCHs, our study corroborates many aspects of the recent literature review by Gaugler and Mitchell (2022). They identified as ways of involvement in residential long-term care: “relationship building with care staff; negotiating with care staff; professional support of staff; management of expectations and the role of families; collaborative engagement with staff; and provision of personal and therapeutic care” (Gaugler & Mitchell, 2022, p. 3). As shown above, we expanded on some of these results presented in the literature review by proving specific examples in the context of EOL care in LTCHs.

Regarding the ways in which care partners want to be involved, we could not identify specific wishes in the context of EOL in a LTCH. Our study shows that those preferences are largely variable. This prompts nurses to pay attention to the behaviors of care partners to deduce how they want to be involved and adjust care accordingly. However, the role of nurses in EOL care goes beyond personalizing care according to their perceptions of care partners’ wishes. They need to assess care partners’ needs and wishes to better offer support (Radbruch et al., 2020). Inferences of this assessment should then be validated.

Despite the limited role played by LTCH health professionals in involving care partners, only a few promotion strategies were suggested by participants, even when prompted with open-ended questions and attempts to stimulate answers. For care partners, this may be explained by their apparent limited knowledge of their potential involvement, as they are unaware of what exactly health professionals can promote. In addition, some nurses viewed their role in the identification of care partners’ wishes for involvement as a passive one of inferring their wishes, rather than one of adopting an active role in assessing them. This may also have influenced their ideas about promotion strategies they could carry out in their clinical practice. The few strategies identified by participants did not indicate that the involvement of care partners is an issue in EOL care. The identified strategies focused on ways to improve EOL knowledge in health professionals and on the contribution of managers to increase that knowledge through training. Although nurses are accountable for keeping their knowledge, skills, and practices up to date with research (Oshodi et al., 2019), this personal responsibility regarding EOL care was not mentioned by participants.

In addition to these strategies, our results described some individual, relational, and organizational characteristics acting as barriers and facilitators to the involvement of care partners. A trust-based relationship was described as fundamental to the EOL experience for both the health professionals and the care partners. When care partners do not trust health the professionals, they act as an advocate to ensure the well-being of the older adult. Jackson et al. (2012) show similar results in their metasynthesis on the perspectives of care partners on EOL in long-term care, while qualifying the active involvement of care partners as hypervigilance. The influencing factors we identified can be useful to guide the development of future interventions aimed at promoting care partners’ involvement. These factors also contribute to the limited body of knowledge on EOL care provided during the last days and hours of older adults in LTCHs.

Recent literature on the COVID-19 outbreak in LTCHs further highlights the importance of involving care partners in EOL care. A critical ethnographic study of care partners who experienced COVID-19 describes harrowing accounts of visitation restrictions, including that of a son who was unable to hold his mother's hand and ensure her comfort during her final days (Bourbonnais et al., 2021b). Care partners felt it was their duty to advocate for the older adult's comfort and safety, and shared feelings of abandoning them when they were unable to do so (Bourbonnais et al., 2021b). This sense of responsibility was particularly evident in our study for one care partner caring for an older adult with a major neurocognitive disorder who had recently been admitted to the LTCH. She actively sought out information from health professionals and monitored the care provided. As recommended by leaders across Canada following the COVID-19 pandemic, care partners are essential to quality of care and should not be considered merely as visitors (Gaugler & Mitchell, 2022).

### Limitations

This study has some limitations. Our sample consisted of seven participants at one LTCH. We had planned to recruit one more care partner, to gain a deeper understanding of the phenomenon, especially with regard to strategies to promote the involvement of care partners, but we had to stop recruiting due to the COVID-19 pandemic. The few strategies suggested to promote the involvement of care partners may be due to the retrospective perspective of all participants, which is another limitation of our study. Still, the participants’ accounts were detailed and allowed us to reach redundancy in data. Also, we did not include the perspectives of health professionals other than nurses, providing EOL care in LTCHs. Furthermore, the care partners who participated in the study were well educated and Caucasian, and two were friends of the older adult at the EOL. These characteristics of the sample reduce the transferability of the results.

### Implications for practice and research

Our findings show the need to promote the involvement of care partners in EOL care to the full extent of its possible scope. To this end, we recommend that LTCH nurses maximize the potential for care partners’ involvement in their day-to-day practice by sharing varied and concrete ways of being involved, by giving them opportunities to ask questions, by actively assessing their wishes, and by avoiding giving superficial information. We also suggest that future research be conducted in LTCHs to develop interventions to facilitate this involvement, as our study identified few strategies.

## Conclusion

In this study, we explored the perceptions of care partners and nurses on care partners’ involvement in the EOL care of an older adult living in a LTCH. We described a possible scope for the involvement of care partners, including information they can obtain and ways they can be present and participate in care. As the wishes of care partners are unique, it is important for nurses who work in proximity with care partners to actively promote how the care partners can be involved in the EOL care of the older adult. Ensuring care partners’ involvement in LTCHs is essential and can optimize the EOL experience of care partners, older adults, and health professionals.
